# Effectiveness of a physical activity intervention program using peer support among sedentary women in Thiruvananthapuram City, India: results of a non-randomized quasi experimental study

**DOI:** 10.12688/wellcomeopenres.16618.1

**Published:** 2021-04-20

**Authors:** Elezebeth Mathews, Odile Sauzet, Kavumpurathu Raman Thankappan

**Affiliations:** 1 Department of Public Health and Community Medicine, Central University of Kerala, Kasaragod, Kerala, 671315, India; 2 Zentrum für Statistik, Universität Bielefeld, Bielefeld, Germany

**Keywords:** Physical activity, women, peer support, India, intervention, Kerala, non-randomized quasi-experimental study, community based

## Abstract

**Background:** Interventions to promote physical activity are very limited in India. The objective of this study was to assess the effectiveness and sustainability of a peer support based physical activity (PA) intervention targeting sedentary women in Thiruvananthapuram City, India **.**

**Methods: **We used a non-randomized quasi-experimental study design with a comparison group. Using the Global Physical Activity Questionnaire (GPAQ) classifications, 401 sedentary women aged 18-64 years were selected by multistage cluster sampling and enrolled into the intervention (n=200) and control (n=201) arms. For the intervention arm, a culturally relevant intervention was delivered to the community stakeholders, participants and peer leaders at three subsequent intensities: intense (three months), less intense (three months) and no intervention (six months). The intervention consisted of a non-communicable disease (NCD) risk assessment, educational workshop, group counselling sessions, goal setting, handbook and peer support. The control participants received printed information on NCDs and their risk factors. PA assessments and anthropometric measurements were made at baseline, 4 ^th^, 7 ^th^ and 13 ^th^ months. Mixed model analysis was done to assess the difference in PA levels between groups at various time points.

**Results:** The proportion of women who were physically active (≥600 MET minutes per week) was significantly higher in the intervention arm compared to the control arm at 4 ^th^ (58.5 % vs 10%, p= 0.001), 7 ^th^ (48.5% vs 6%, p= 0.001)) and 13 ^th^  month (29.6 % vs 0.6%, p =0.001), respectively. Improvements from baseline PA expended by the intervention arm compared to the control arm in MET-min / week were 990, 575, and 466 at 4 ^th^, 7 ^th^ and 13 ^th^ months, respectively.

**Conclusions:** A PA intervention using peer support was found to be effective among women in India. Improvements in PA in the intervention arm decreased over time particularly after the no-intervention phase indicating the need for integrating it with community organizations.

## Introduction

Physical inactivity accounted for 1.3 million deaths in 2017 globally ( GBD, 2017). Physical inactivity, a major risk factor for death and disability due to non-communicable diseases (NCDs) ( Lee *et al.*, 2012) has been found to be higher among women (33.9 %) than in men (27.9 %) worldwide ( Bauman *et al.*, 2012). In Kerala, the most advanced Indian state in epidemiological transition, women were reported to have higher prevalence of NCDs and risk factors such as physical inactivity ( Shah & Mathur, 2010; Thankappan *et al.*, 2010) and overweight ( IIPS, 2008). Culture and gender norms have restricted women from engaging in leisure time physical activity (PA) such as brisk walking and other moderate intensity sports ( Mathews *et al.*, 2016). A declining trend in physical activity at work and transportation was reported ( Shah & Mathur, 2010) among women due to increased mechanization and urbanization.

 Promotion of physical activity through community-based activities using informational, behavioral, social, policy and environmental approaches have been well advocated and reported to be effective in developed nations ( Baker *et al.*, 2011). Interventions using informational approaches have used point of decision prompts ( Brownell *et al.*, 1980), community wide ( Roux *et al.*, 2008) and mass media campaigns ( Bauman *et al.*, 2001). Interventions using behavioral and social approaches have used individually adapted stage targeted interventions ( Marcus *et al.*, 1998), behavioral modification education ( Calfas *et al.*, 2000), behavioral modification counselling ( Glasgow *et al.*, 2001), physician based counselling ( Calfas *et al.*, 2002), telephonic counselling ( Green *et al.*, 2002), web based counselling ( De Vries & Brug, 1999), social support interventions ( Amorim *et al.*, 2010) and family/home based interventions ( Stephens *et al.*, 1985). Interventions using the environmental approach have focused on creating or enhancing access to places for physical activity ( Mohan *et al.*, 2006), use of community scale urban design and land use policies ( Aytur *et al.*, 2007), transportation policy and infrastructure change ( Aytur *et al.*, 2007) and community wide policies and planning ( Hoehner *et al.*, 2008).

Most of these interventions were developed and implemented in high resource settings where there was adequate infrastructure and systems to support behaviour change, with information and support provided on a one to one or group basis laying the responsibility of making a behavior change on the individuals themselves, whether it was diet, physical activity or quitting alcohol and tobacco. However, in developing countries such as India, where the systems support in terms of health professionals, health systems and socio-environmental conditions are very limited, an individualistic approach to behavioral management is quite challenging and difficult to sustain.

Evidence suggests that support from friends, neighbors, and spouses play a crucial role in being physically active ( Mathews *et al.*, 2015). Recent advances in chronic disease care and management show that peer support enables people to share their experiences and provides the practical, emotional, and ongoing support that are critical to sustained behavior change ( Boothroyd & Fisher, 2010). Several studies have utilized the concept of peer support in diabetes management ( Philis-Tsimikas *et al.*, 2004), arthritis care ( Barlow *et al.*, 2000), mental health ( Davidson *et al.*, 2006) and self-directed behavior change ( Keyserling *et al.*, 2002). In India, peer support has been found to play an important role in chronic disease management ( Aswathy *et al.*, 2013) and could be considered as a viable strategy to promote physical activity among women in India as social support from peers plays a crucial role in overcoming the individual level constraints and barriers related the gender and cultural norms ( Eger *et al.*, 2018).

Research on physical activity interventions is limited in India. Intervention studies have mostly focussed on lifestyle modification, specifically diabetes prevention ( Ramachandran *et al.*, 2001; Satish *et al.*, 2013). However, there have been no intervention studies reported from India with the primary aim of promoting physical activity among adults, specifically women. Given the higher risk for women in developing chronic diseases and the inherent nature of women having fewer opportunities to be active, an intervention trial was conducted to promote physical activity among sedentary women in Thiruvananthapuram city, using peer support. The objective of the study was to assess the effectiveness and sustainability of a culturally specific intervention using peer support in increasing the proportion of physically active women in the intervention arm when compared to the control arm after one year of intervention.

## Methods

### Ethical approval

Ethical approval for this study was obtained from the Institute Ethics committee of Sree Chitra Tirunal Institute for Medical Sciences and Technology, Trivandrum (approval number: SCT/IEC/383/NOVEMBER-2011 dated 28.11.2011) and have been performed in accordance with the ethical standards as laid down in the 1964 Declaration of Helsinki and its later amendments or comparable ethical standards. The trial was registered in the Clinical Trials Registry of India (CTRI registration number: CTRI/2011/12/002222 [registered on 13.12.2011]). Written informed consent was obtained from all individual participants included in the study.

### Study settings

Thiruvananthapuram district of Kerala has a similar human development index and literacy rate to that of the state as a whole ( Government of Kerala, 2005). This study was conducted in the expanded part of Thiruvananthapuram City which constitutes the five erstwhile “ *Panchayats*” (lowest administrative unit of local self–government) added to the city corporation in the year 2010 due to their proximity to the city and other developmental activities in these regions. These five erstwhile *Panchayats* were chosen for the study due to feasibility. The newly added erstwhile *panchayats* constituted 14 wards of the 100 wards in the Thiruvananthapuram City Corporation.

### Study design

This study followed a non-randomized quasi- experimental study design with a comparison group. The intervention was for a period of 6 months with 3 months of intense phase and another 3 months of less intense phase. Multi- level engagement of varying intensities was delivered targeting community, participants and peer leaders. At the community level, the office bearers of residents’ associations and self-help women’s group were briefed on the need and nature of the intervention, and sought support, both for logistics and structural changes. Study participants were engaged in individual NCD risk assessment through a medical camp, educational workshop, group counselling sessions, peer leader led sessions for goal setting and goal review, participant handbook and guided culturally appropriate activities of choice such as group /individual walking and/or aerobic dance sessions. A peer leader, amongst the participants in each cluster was identified, trained and given ongoing support for group mentoring. Intervention at all the levels were delivered of varying intensity ( Table 1). The control participants received printed information materials on non-communicable diseases and their risk factors.

**Table 1.** Phases of the intervention.

Level of intervention	Intense phase	Less intense phase	No intervention phase
Community	✓ Community mobilization ✓ Engagement with the stake holders ✓ Community ownership of the program	✓ Participation in community events such as annual day ✓ Advocacy at events	✓ Community initiatives for sustainment
Participants	✓ Individual NCD risk assessment through medical camps: assessment of waist circumference, body mass index and blood pressure. ✓ Educational workshop ✓ Group counselling ✓ Goal setting and goal review ✓ Participant handbook ✓ Group walking ✓ Aerobic dance sessions	✓ Goal setting and review ✓ Peer leader led meetings within the group ✓ Supporting and motivating the participants to sustain the behavioral change ✓ Continuing group-based activities	✓ Self-monitoring of the behavior
Peer leaders	✓ Peer leader selection ✓ Peer leader training and capacity building to assist the group in behavioral change ✓ Peer leader and participant workbook	✓ Organizing meeting within and outside the group ✓ Organize walking groups ✓ Support the participants in making behavioral change	

### Sample size estimation

Sample size was estimated with the anticipated assumption that 30% ( Prince *et al.*, 2014) of participants in the intervention arm would meet the physical activity recommendations compared to 10% in the control arm after the intervention trial. In order to detect the 20% difference in the proportion of people achieving the WHO PA recommendations (at least 150 minutes of moderate-intensity aerobic PA throughout the week or at least 75 minutes of vigorous-intensity aerobic PA throughout the week, or an equivalent combination of moderate- and vigorous-intensity activity) (WHO GPAQ) between the intervention and control arm with a confidence level of 95% and a power of 80%, the sample size required was 94 ( Smith & Morrow, 1996). Considering a loss to follow up of 6% and a design effect of two for the cluster sampling, a sample of 200 in each arm (n=400) was to be enrolled into the trial. Based on an anticipated physical inactivity prevalence of 31.8 % among women aged 15–64 years ( Satish, 2008), the survey had to be conducted among at least 1258 women [(100/31.8) *400=1258)] in order to get 400 sedentary women for the intervention trial.

### Sample selection process

**Figure 1.**
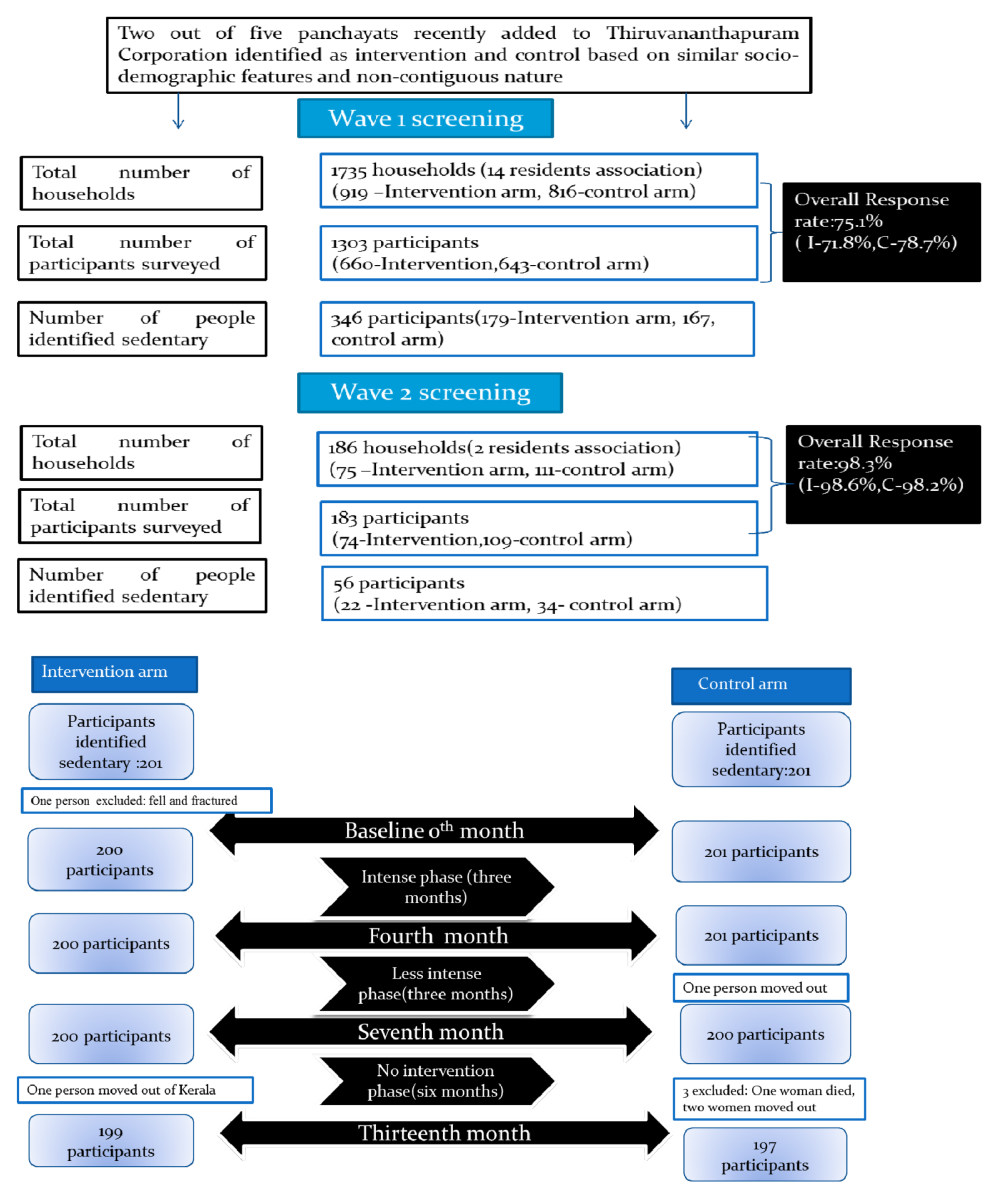
Schematic representation of the selection of the study sample.

The sample consisted of sedentary women. The sample selection scheme is shown in Figure 1. The sample was selected from the residents’ associations of the two selected panchayats, which enrolled over 95% of the households. Each residents’ association had a number of households varying from 25 to 250 with an average number of 93. Out of 71 residents’ associations in these two panchayats, 14 (1258/93=14) were randomly selected through random number generator in Microsoft excel (seven each in the intervention and control arms) initially in order to get the sample size of 1258 women. An additional two residents’ associations were similarly selected to ensure an adequate sample for the intervention trial.

Sedentary women for this trial was identified through a house to house survey using a structured interview schedule by the trained data collectors ( Mathews *et al.*, 2015). The interview schedule consisted of socio-demographic information, self-reported physical activity assessed through Global Physical Activity Questionnaire (GPAQ), and factors influencing physical activity. All households within the residents’ association were surveyed. Those women who consented were included in the survey. Women with physical deformities, those who were bedridden, those who were pregnant and lactating, those who were less likely to reside in the area for the next 6 months, and those with disease conditions where PA was contraindicated were excluded from the survey. In wave 1 screening, 1303 participants were identified from 1735 households and in wave 2 screening, 183 participants were surveyed from 186 households. Locked houses were excluded if they remained locked the second time of visit, and in instances of more than one eligible woman per household, one was selected randomly. We identified 402 women as sedentary. All women identified as sedentary and willing to participate in the trial were subsequently enrolled.

### Data collection techniques

Data collection and entry were done by trained voluntary workers at the community using an interview schedule. Data captured at the household included socio-demographic information, physical activity assessment, and factors influencing PA at individual, family and community level ( Mathews *et al.*, 2015). Physical activity level of the women was assessed using the Global Physical Activity Questionnaire (GPAQ), validated in India ( Bull *et al.*, 2009). GPAQ captures physical activity from three domains - work, transport and leisure. Women with a total physical activity level of <600, 600–2999, and ≥3000 metabolic equivalent task (MET) minutes per week were classified into low (sedentary), moderate, and high PA levels respectively ( Bull *et al.*, 2009).

At the baseline, apart from the self-reported physical activity, anthropometric assessments including height, weight and waist circumference were taken at the NCD risk assessment mobile clinic organised in each residents’ association by two trained medical professionals (a medical doctor and the PI, who is a trained nurse). Anthropometric measurements were taken using standard equipment and protocol ( WHO, 2005). 

Similarly, physical activity as measured by the GPAQ, physical activity behaviour, and facilitators and barriers in PA engagement were made at fourth, seventh and thirteenth month of intervention. At the fourth and thirteenth month of intervention, anthropometric measurements were taken at the individuals’ household by the same data collector. Data collectors were blinded to the study groups.

### Intervention development

Intervention development adhered to the intervention mapping protocol ( Bartholomew *et al.*, 1998). The physical activity goal was derived from the WHO global strategy for diet and physical activity ( WHO, 2014). Intervention components were identified based on the findings from the formative research which included focus group discussions ( Mathews *et al.*, 2016) and a cross-sectional survey ( Mathews *et al.*, 2015), and was supported by some theories of health promotion. In this previous research, focus group discussions among women residents captured the perceptions on barriers and facilitators of physical activity. Lack of knowledge/ awareness on the physical activity recommendations, benefits of PA, activities to engage and misperceptions on the intensity of activities were key findings. Participants also stated that they need company to engage in any outdoor PA. The positive correlates of PA from the cross-sectional survey were knowledge on benefits of PA and support from friends and neighbours.

Based on the findings, intervention aimed to address the gap in knowledge through educational workshop, increase self-awareness on the impending risk associated with the sedentary lifestyle through NCD risk assessment mobile camps, enhance social support through group counselling and peer support, and engage in sustainable behaviour change through application of behavioural theories of reasoned action and planned behaviour, social cognitive theory, enhancing self-efficacy and peer support.

**Figure 2.**
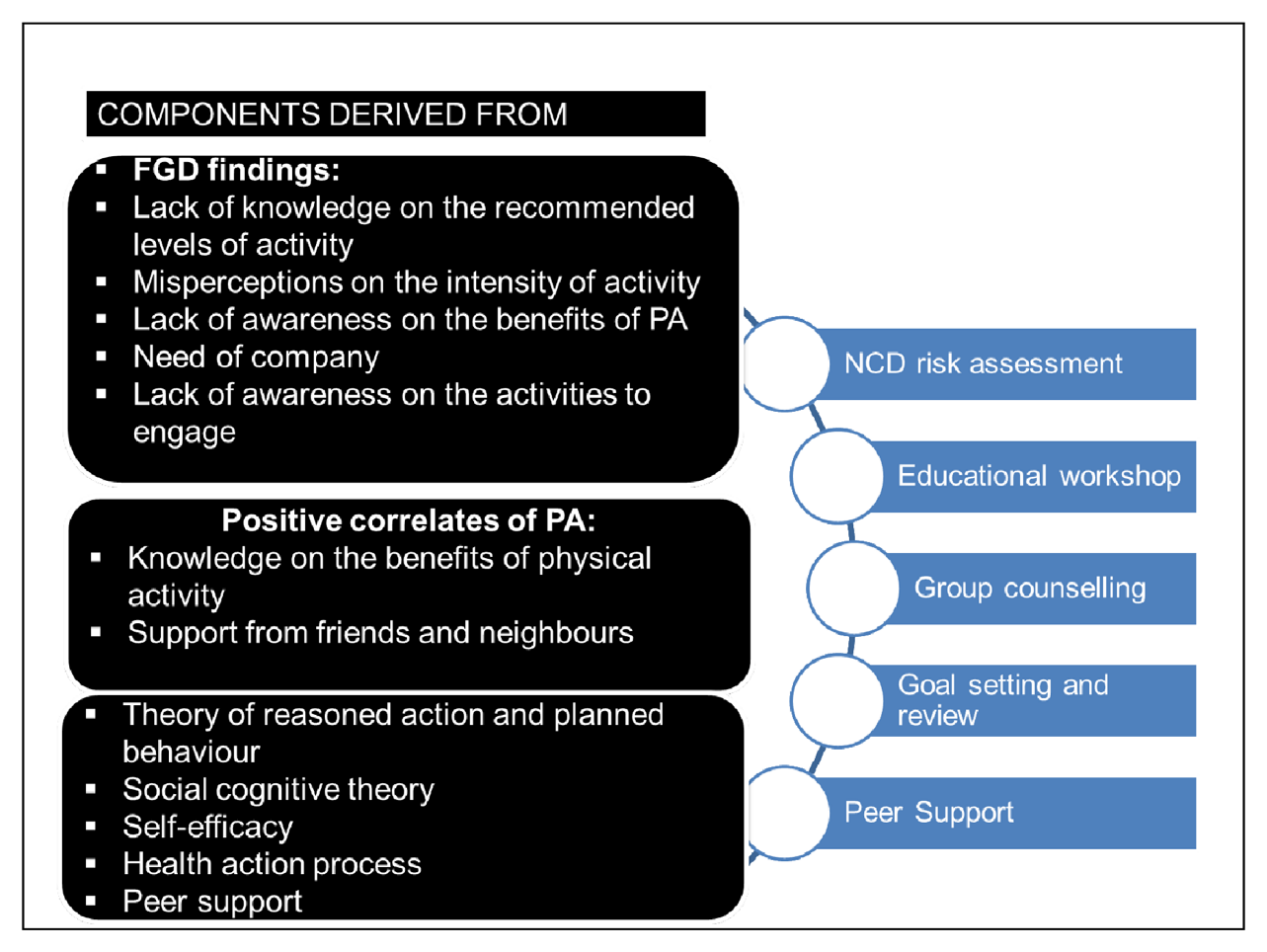
Processes involved in the identification of the intervention components.

Figure 2 describes the processes that lead to the identification of the intervention components.

### Phases of intervention delivery at multiple levels

Informed written consent was obtained from the participant at the baseline survey for participation in the study. Subsequent to being identified as sedentary, their willingness to participate in the trial was sought. Among 402 women identified as sedentary, 401 women consented to further participate in the trial.

Table 1 shows the phases of intervention with activities at different levels - community, peer leader and participants. The intervention was delivered in three phases: the first three months of an “intense” phase, the next three months of a “less intense” phase and the latter six months of a “no intervention” phase. 

  a. Intense Phase of Intervention (0-3 months)

At the community level, activities were focused on community mobilization, which involved, preliminary stakeholder meetings with the local women’s self-help group *(Kudumbashree*) and the office bearers of residents’ association for rapport, support and ownership of the program. This has led to strong support in facilitating the organization of mobile camps for NCD risk assessment in the neighbourhood and conduct of regular peer led meetings. Subsequently, discussions were held on making the local area physical activity friendly to overcome the stray dog menace and converting “dead use” land to walking paths. 

At the participant level, activities included NCD risk assessments, educational workshops and peer group meetings within each cluster. Women identified as sedentary had NCD risk assessment at the mobile camps organised in their neighbourhood, where blood pressure and anthropometric assessments were done. On the same day, an educational workshop was conducted to raise awareness on NCDSs, physical activity, its benefits and the recommendation for health benefits.

Participants were provided with a handbook which consisted of two Units. Unit 1 focussed on physical activity and health, and included the following contents: importance of physical activity, change in the lifestyle over generations, types and benefits of physical activity, role of physical activity in preventing premature mortality, diabetes mellitus, cardiovascular diseases, stroke and other non-communicable diseases, improved mental health, and recommendations of PA for health benefits by World Health Organization (WHO). Unit 2 focussed on practical components to assist the participants in behaviour change. It included locally relevant activities of moderate-vigorous intensities, identifying facilitators and barriers, goal setting, mapping the locality for activities that are pursuable, and goal reviews in peer leader meetings. Regular fortnightly meetings with the women participants were organised in the households of these women members to discuss on the progress of achieving individual level physical activity goals. Within each residents’ association, a peer leader was identified and trained for five days on mentoring, communication skills and contents of the peer leader handbook. The initial three meetings were led by the PI in the presence of the peer leaders. Subsequently, the peer leaders conducted the meetings and contacted the Principal Investigator to update on the progress of the meetings. The average participation rate was 70% in the intense phase of intervention.

The less intense phase involved peer leader led sessions to support and motivate participants to sustain physical activity behaviour change, with no PI involvement. During the no intervention phase, no active engagement was made with the intervention groups. However, informal gatherings took place.

Participants in the control arm were provided with educational booklets on chronic diseases at the baseline and no engagements were made thereafter apart from the 4 ^th^, 7 ^th^ and 13 ^th^ month assessments. All study materials can be found as extended data ( Mathews, 2021).

### Variables

The outcome variable is the physical activity measured using GPAQ quantified in MET minutes per week. The independent variables were age (completed years), educational status, occupation, marital status and anthropometric measures such as height, weight and waist circumference. Participants were asked to report on the activities undertaken to achieve the recommended levels of activity, the facilitators and barriers of physical activity. Participants were asked to rate the aspects of intervention that motivated to take up the activity such as peer supporters, group counselling, support from family, support from neighbours, being part of the group and the information booklet.

### Data analysis

Data were analyzed using STATA version 11.2 and SPSS version 17. Data from the GPAQ were scored using SPSS version 17 (IBM Corp). The GPAQ data on physical activity were quantified as MET minutes per week and were derived by adding the products of intensity (MET), duration (minutes) and frequency (number of times per week) of each of the reported work-related, travel-related, and leisure-related physical activities. A value of 4 METs was assigned for moderate intensity activity and a value of 8 METs was assigned for vigorous intensity activity. Domain specific scores on work, transport and leisure were calculated to understand the changes made in each domain. Individuals with missing data were excluded from the analysis.

The chi-square test was done to examine whether the baseline parameters such as age, educational status, occupation and marital status were significantly different between the intervention and control arms. Multilevel mixed model analysis was done to assess the intervention effectiveness in terms of physical activity energy expenditure during the study period after considering the effect of time, clusters and groups, and considered the non-independence of observations within patient and patient within clusters. Longitudinal data analysis considered individual, cluster, groups and time point at four levels. Study participants were referred to as individuals. Cluster was referred to the cluster sampling design adopted in the study and there were eight clusters each in the intervention and control arm. Group referred to the intervention and control arms of the intervention trial. Time referred to in the model was the multiple time points at which assessments were made namely baseline, fourth, seventh and thirteenth months. The effect of the intervention was analysed at two levels in the model. The difference in the mean level of physical activity between the intervention and the control arm at each time point was estimated using time-group interaction with time being a dummy variable. The improvement in physical activity level from baseline due to effect of intervention at each time when compared to the control arm was estimated using time-group interaction with time as a continuous variable ( Sauzet *et al.*, 2015). The effect of confounding for age, educational status, occupational status and marital status was eliminated in the analysis by including those variables in the model with the level of significance at ‘p’ value of 0.1 between the intervention and control arm. Distribution of age and occupation was significantly different between the intervention and control arms with a ‘p’ value of 0.1 and hence was included in the model.

## Results

A total of 401 women (intervention - 200, control - 201) were enrolled in the trial at the baseline. At the end of 13 ^th^ month, five women dropped out. The detailed participant flow chart is given in Figure 1. The mean age of the study participants in the intervention arm was 48 years (SD 0.72) and 46 years (SD 0.86) in the control arm. Table 2 describes the baseline characteristics of the study sample population ( Mathews, 2021).

**Table 2.** Baseline characteristics of the sample.

Categories	Intervention arm (n=200) N (%)	Control arm (n=201) N (%)	P value *
**Age group (years)**			
**<35**	025(12.5)	047(23.4)	0.001
**35– 54**	117(58.5)	086(42.8)	
**55+**	058(29.0)	068(33.8)	
**Education status**			
**Up to high school**	104(52.0)	100(49.7)	0.6
**Higher secondary and above**	096(48.0)	101(51.3)	
**Current Occupational status**			
**Employed**	031(15.5)	051(25.4)	0.01
Unemployed **	169(84.5)	150(74.6)	
**Marital status**			
**Married**	179(89.5)	170(84.6)	0.2
Others ***	021(10.5)	031(15.4)	

*Chi square p value comparing the proportion between intervention and control arm.

**Housewives, retired, unemployed and students, ***Unmarried, separated, divorced and widowed

Table 3 describes the physical activity pattern of study participants at multiple time points. At the 4 ^th^ month, after intense phase of intervention, 58.5% of women were found to be active in the intervention arm compared to 10% active women in the control arm (p value-0.001). At the seventh month, subsequent to a three month less intense phase of intervention, 48.5% of women were found to be active in the intervention arm compared to 6% in the control arm. With no intervention for a period of six months, the proportion of active women was 29.6% in the intervention arm compared to 0.6% in the control arm.

**Table 3.** Physical activity pattern over multiple time points.

Time point	Activity levels	Number in the intervention arm	Intervention arm N (%)	Number in the control arm	Control arm N (%)	P value *
Baseline	Inactive	200	200 (100)	201	201 (100)	
	Active		000 (000)		000 (000)	
4 ^th^ month	Inactive	200	083 (41.5)	201	180 (90.0)	0.001
	Active		117 (58.5)		021 (10.0)	
7 ^th^ month	Inactive	200	103 (51.5)	200	188 (94.0)	0.001
	Active		097 (48.5)		012 (06.0)	
13 ^th^ month	Inactive	199	140 (70.4)	197	196 (99.4)	0.001
	Active		059 (29.6)		001 (00.6)	

*Chi square p value comparing the proportion between intervention and control arm.

Table 4 describes the mean levels of physical activity, body mass index (BMI) and waist circumference in both intervention and the control arms at multiple time points.

**Table 4.** Mean levels of physical activity and anthropometric parameters at multiple time points.

Time point	Parameters	Number of women in the intervention arm	Intervention arm Mean (SD)	Number of women in the control arm	Control arm Mean (SD)
Baseline	Body mass index (kg/m ^2^)	200	26.96 (3.80)	201	26.72 (5.11)
	Waist circumference (cm)	200	89.81 (9.09)	201	87.90 (11.64)
	Physical activity level (MET minutes per week)	200	84.30 (152.51)	201	120.60 (179.99)
4 ^th^ month	Body mass Index(kg/m ^2^)	200	26.85 (3.78)	201	26.85 (5.12)
	Waist circumference (cm)	200	89.62 (8.97)	201	88.04 (11.62)
	Physical activity level (MET minutes per week)	200	1159.22 (1065.60)	201	202.19 (314.00)
7 ^th^ month	Physical activity level (MET minutes per week)	200	707.30 (608.43)	200	168.26 (310.34)
13 ^th^ month	Body mass Index(kg/m ^2^)	199	27.79 (4.08)	197	27.09 (5.15)
	Waist circumference(cm)	199	89.22 (11.22)	197	88.09 (11.37)
	Physical activity level (MET minutes per week)	199	525.93 (547.97)	197	95.12 (156.63)

MET=metabolic equivalent task; SD=standard deviation

Women rated group counseling, social support from family, and peer support as important intervention components that motivated for being active. Table 5 shows the modelled estimate of physical activity level in the intervention and control arms at fourth, seventh and thirteenth month. The coefficient of change was the amount of physical activity expended at each time point when compared to the baseline. The coefficient of change (MET minutes per week) from baseline was significantly higher in the intervention arm compared to the control arm at 4 ^th^ month (1075.02 vs 82.96), 7 ^th^ month (623 vs 47.68) and thirteenth month (441.02 vs -25.45), p=<0.001.

**Table 5.** Modelled estimate of physical activity level in the intervention and control arms at multiple time points.

Time point	Intervention arm		Control arm	
	Coefficient of change from baseline (MET minutes per week) (95% CI)	Mean physical activity * (MET minutes per week) (95%CI)	Coefficient of change from baseline (MET minutes per week) (95% CI)	Mean physical activity * (MET minutes per week) (95%CI)
4 ^th^ month	1075.02 ** (952.66-1197.38)	1158.90 (942.96-1375.08)	82.96 (43.47-122.46) **	203.36 (129.2-277.67)
7 ^th^ month	623.00 ** (500.67-745.32)	706.97 (490.92-923.02)	47.68 (08.24 - 87.12) *	168.08 (93.97-242.19)
13 ^th^ month	441.02 ** (318.53-563.51)	524.99 (308.77- 741.21)	-25.45 (-64.89-13.98)	094.95 (20.84-169.05)

Variables in the model: Individual, intervention arm, cluster, and time point. MET=metabolic equivalent task; CI=confidence interval

* Estimated from the model based on the linear mixed effect model

**P value for the coefficient of change from the baseline significant at <0.001 level

* P value for the coefficient of change from the baseline significant at 0.01 level

**Figure 3.**
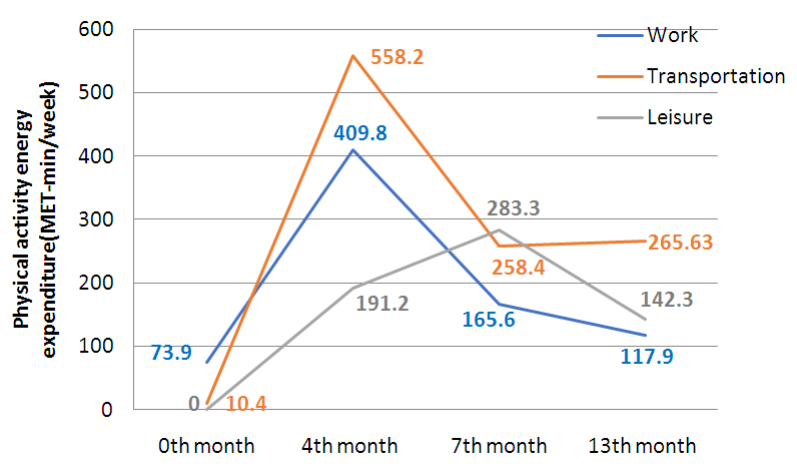
Domain specific progress in the intervention arm at multiple points.

Domain specific progress in the intervention arm at multiple points ( Figure 3) were analysed. Much of the physical activity (MET min/week) reported after the intense phase of intervention was in transportation and work compared to leisure (558.2 vs 409.8 vs 191.2). However, at the 7 ^th^ month, leisure time PA was reported the highest (283.3) compared to transportation (258.4) and work (165.6).

**Figure 4.**
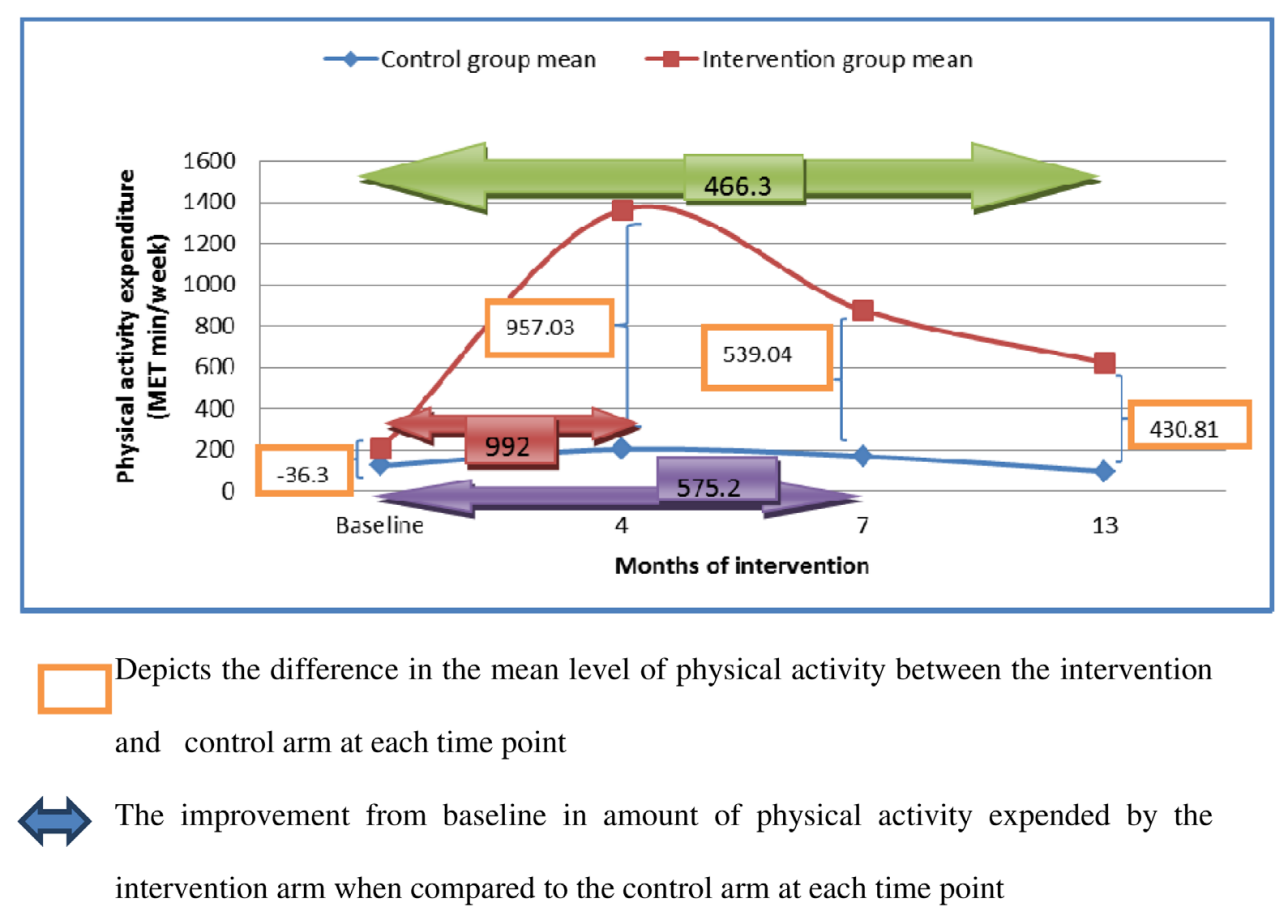
Modelled estimate of physical activity level in the intervention arm compared to the control arm at multiple time points. The orange boxes depict the difference in the mean level of physical activity between the intervention and control arm at each time point. The horizontal arrows show the improvement from baseline in amount of physical activity expended by the intervention arm when compared to the control arm at each time point.

Figure 4 represents the modelled estimate of physical activity level in the intervention arm compared to the control arm at multiple time points. At the fourth month of intervention, women in the intervention arm expended 1075 (95% CI: 952.66-1197.30) MET-min / week more than at baseline. The energy expended was 623 (95% CI: 500.67-745.32) MET-min/ week more in the seventh month of intervention when compared to the baseline. At the thirteenth month of intervention, the energy expended by the women in the intervention arm was 441 (95% CI: 318.53-563.51) MET-min/ week more when compared to the baseline.

The analysis showed that the intervention arm had more physical activity compared to the control arm at all the three time points: at month four, 992 (SD: 65) MET minutes per week more than the control arm, at month seven, 575 (SD: 65) MET minutes per week more than the control arm and at month 13, 466 (SD: 65) MET minutes per week more than the control arm.

## Discussion

Promotion of physical activity among sedentary women in Thiruvananthapuram city using peer support was found to be effective during the study period. The highest proportion of active women during the study period was after the three months’ intense phase in the intervention arm when compared to the control arm (58.5% vs 10%). This could be due to the intense nature of the intervention. The educational workshop sensitized the women on several dimensions such as recognizing the importance of physical activity, intensities of activity needed for health benefit especially for chronic disease prevention, breaking the misperception of adequacy of physical activity from household work and setting up realistic and feasible goals with the support of group members. Group based activities including walking groups have also been found to be beneficial in other studies where a walking program among elderly cancer survivors showed that the proportion of participants walking 150 minutes per week increased from 21% at the baseline to 50% over a six-month period ( Nyrop *et al.*, 2014).

The proportion of active women decreased gradually after the “intense” phase, based on the assessment made at the seventh month; however, the number of participants who remained active in the intervention arm was significantly higher than the control arm (48.5% vs 6 %). The decrease could be either due to the reduced intensity of the intervention or reduced sustainability of intervention over time. However, it is supposed that the monthly meetings with the peer leader and peer support from the group-based activity would have facilitated in sustaining the activity among active women. The proportion of active women in our study after six months of intervention (48.5%) was lower than another lifestyle intervention study (78%) in the United States (US) which involved group-based meetings and goal settings ( Dunn *et al.*, 1998). Apart from the group-based activities and goal setting, increased access to facilities in the US could have played a role in higher proportion being active after the intervention, than in our setting.

The latter six months of “no intervention” period showed a reduction in the proportion of active women to half (29.6%) of the intense phase (58.5%). A physical activity promotion study which targeted both sedentary and physically active participants had shown that participants who were already active were more likely to adhere to the intervention and maintain a healthy lifestyle than the sedentary or inactive ones ( Bock *et al.*, 2001). The relative proportion of active women was higher in the intervention arm compared to the control arm (29.6% vs 0.6%) despite the reduced proportion of active women in the intervention arm with the tapering dose of intervention. The multiple strategies of health behavior theory adopted in this study such as improving self-efficacy through goal setting, goal review, self-monitoring and peer support along with support from family and friends would have facilitated the positive behavior change and its maintenance, as reported in another study ( De Greef *et al.*, 2011). Apart from increased self-efficacy through the educational workshop and individualized counselling, social support from peers and family would have played an important role in the sustenance of activity. Group based intervention delivery has been reported as effective among women and more effective than individual or community-based interventions ( Cleland *et al.*, 2013). The effect of an intervention may further decrease over time and it is important to devise strategies for periodic prompts and integrate them with existing community-based initiatives.

An increase in the proportion of active women in the control arm could be due to the influence of the educational booklets given to them. The decline in physical activity in the control arm below the baseline level at thirteenth month suggests that with no intervention, individuals tend to go back to their initial sedentary state or retrograde further. This calls for the need to initiate physical activity promotion activities in the community in the wake of rising non-communicable diseases and their risk factors ( Jeemon *et al.*, 2019).

A majority of the women who became active at fourth, seventh and thirteenth months of intervention were mostly engaged in moderate intensity activity. Studies have shown that women prefer to do moderate intensity activity over vigorous ones ( Forbes, 2014) and walking as the most preferred activity ( Bélanger *et al.*, 2012). Domain specific mean levels of physical activity suggests that during the intense phase, most changes were made in the transportation domain (558.2 MET min/week) followed by work (409.8) and leisure (191.2). There was as an increase in the moderate intensity activities reported at domestic work. This could be because the majority (84.5%) of them were unemployed and housewives. The increased physical activity level in the transportation domain was because most of them chose to walk for shopping and work. Leisure time activities involved walking individually or in groups and aerobic dancing sessions. Not only leisure time physical activity but physical activity in domains particularly transport and work have been found to have numerous health benefits ( Samitz *et al.*, 2011).

A dose–response relationship between the intervention and physical activity was found in this study, similar to another study ( Rodondi *et al.*, 2006) and the sustained positive behavior change even in the no intervention phase could be due to the effect of social and peer support. A meta-analysis on physical activity promotion interventions targeting women revealed that the mode of intervention delivery was the key factor that determined the intervention’s effectiveness. Group based interventions proved more effective in achieving the targets than individually tailored interventions ( Cleland *et al.*, 2013).

Group counselling and information booklets were consistently reported by women as facilitators for physical activity in this study which stresses the importance of information dissemination and assistance in goal setting for making physical activity choices. A previous study reported that goal setting and counselling were important intervention component for behavior change ( Ries *et al.*, 2014). Sufficient evidence on the effectiveness of counselling by doctors or health care providers in improving physical activity is available ( Wattanapisit *et al.*, 2018). However, in countries such as India, with limited health system resources including health care providers such as doctors, it is imperative to render the services of a peer leader in the community for activities of health promotion. As behavior change needs constant assistance and support for adoption and maintenance ( Kwasnicka *et al.*, 2016), it is only practical to engage the community with effective use of peer support.

The culture of PA and sport among women in South India differs markedly from that in regions with the most success in PA promotion such as Brazil, US, Australia and Europe which have invested in social capital and the environment for physical activity promotion. A recent study from Brazil reported a decline in age standardized mortality attributed to physical inactivity which could be due to the improvement in physical activity in the country ( Silva Das *et al.*, 2020). In order to bridge this gap, it may require concerted action over a period of time, through government and non-governmental organizations to facilitate transferability and better uptake of the interventions.

A limitation of our study was that it was done in the expanded part of the Thiruvananthapuram city and hence the results may not be generalizable to the entire city. Another limitation was that physical activity was assessed using the GPAQ which is self-reported and not validated with objective measures such as accelerometers or pedometers. However, self-reports have been recommended for epidemiological studies with sufficient validity and reliability ( Bull *et al.*, 2009)

Our study points out that “single strategy” does not assist women in making a behavior change. Multiple tailored strategies will have to be employed at multiple time points at multiple levels, i.e. improving personal motivation and self-efficacy at personal level, support from family, spouse and peer support at the interpersonal level, and conducive environment for active living at the community level. High political commitment with mobilization of resources is required for addressing physical inactivity described as a global pandemic in a lancet series on physical activity ( Kohl *et al.*, 2012)

## Data availability

### Underlying data

Open Science Framework: Peer support interventions to promote physical activity among sedentary women, India. https://doi.org/10.17605/OSF.IO/CK2AP ( Mathews, 2021).

This project contains the following underlying data:

- Raw dataset of participant’s reported physical activity levels, at baseline, fourth, seventh and thirteenth month of the intervention, as well as anthropometric parameters and intervention components adopted at relevant time points.

### Extended data

Open Science Framework: Peer support interventions to promote physical activity among sedentary women, India. https://doi.org/10.17605/OSF.IO/CK2AP ( Mathews, 2021).

This project contains the following extended data:

- Intervention material developed in the vernacular language (Malayalam)
- Consent form (in both Malayalam and English)
- Data collection tool (in both Malayalam and English)
- Variable description

Data are available under the terms of the Creative Commons Attribution 4.0 International license (CC-BY 4.0).
