# Supplementary material for: Effectiveness of a physical activity intervention program using peer support among sedentary women in Thiruvananthapuram City, India: results of a non-randomized quasi experimental study
Source: Wellcome Open Res. 2021 Apr 20;6:87. [Version 1] doi: 10.12688/wellcomeopenres.16618.1 (PMC8145223; doi:10.12688/wellcomeopenres.16618.1)
Supplement: Peer review history

# Peer review: Reviewer response for version 1

**DOI:** 10.21956/wellcomeopenres.18321.r43732

**Published:** 2021-06-01

**Authors:** Aswathy Sreedevi

**Affiliations:** 1 Department of Community Medicine, Amrita Institute of Medical Sciences, Kochi, Kerala, India

The paper by Elezebeth Mathews *et al.* is a well written document of the outcomes of an intervention on improving physical activity which has been diligently executed. Current literature has been cited appropriately. The study design is appropriate. Sufficient details of the methods are provided. The statistical analysis is appropriate.

- The intervention consisted of a NCD risk assessment, educational workshop, group counselling sessions and goal setting. The authors report a significant improvement in physical activity in the intervention arm at 4th and 13th month. It is a complex intervention at multiple levels.
- The intervention was delivered at the cluster level and the outcomes are measured at the individual level which can lead to some bias, though it may be minimal due to a similar demographic distribution of the women. Did the sedentary only attend the NCD risk assessment camp?
- The timeline for the conduct of the study does not take into account the duration of the baseline, midline and endline surveys.
- Any particular reason why the response is greater in the second wave of screening? The drop out rate is next to negligible in both the arms. It would also be interesting for the readers to know if there is any particular reason for this.
- In intervention development, “Lack of knowledge/awareness on physical activity recommendations, benefits of PA, activities to engage…were key findings…" can the authors be more specific on this?

It would be good if tables 3 and 4 are simplified and repetition avoided. For example, Table 4 could be written as:

                                                  Intervention                                                    Control

                         0          4          7          13months                    0          4          7          13months

 BMI              26.96       26.85   -           27.79                      26.72         26.85   -           27.09

  

- Table 5 mentions the coefficient of change - what is presented in the table seems to be absolute difference in met minutes/week from baseline to 4 ^th^ month, baseline to 7 ^th^ month etc.
- Are there any unintended effects of the intervention? This should also be mentioned.
- In the abstract, in conclusion it is mentioned that physical activity can be integrated with community organisations - this is not very clear.
- The multiple levels of intervention make it difficult to assess which intervention is the most effective and can be a limitation too.

I would recommend this paper for acceptance as it is a logical and cohesive piece of work carried out scientifically to improve physical activity.

Is the work clearly and accurately presented and does it cite the current literature?

Yes

If applicable, is the statistical analysis and its interpretation appropriate?

Yes

Are all the source data underlying the results available to ensure full reproducibility?

Yes

Is the study design appropriate and is the work technically sound?

Yes

Are the conclusions drawn adequately supported by the results?

Yes

Are sufficient details of methods and analysis provided to allow replication by others?

Yes

Reviewer Expertise:

Non Communicable Disease and Cancer screening

I confirm that I have read this submission and believe that I have an appropriate level of expertise to confirm that it is of an acceptable scientific standard.

# Peer review: Reviewer response for version 1

**DOI:** 10.21956/wellcomeopenres.18321.r43731

**Published:** 2021-05-24

**Authors:** P. Prasanna Mithra

**Affiliations:** 1 Kasturba Medical College Mangalore, Manipal Academy of Higher Education, Manipal, Karnataka, India

This non-randomized quasi experimental study has a strong literature back up. Methods have been spelled out adequately and there is clear explanation of execution of the interventions across two arms. 

 Plus point of this study is considering the natural living conditions of study participants and making these interventions "culture friendly". This would enhance the acceptability of these interventions in real world situations in future. 

 One challenge in generalizing the findings would be in the relatively different age group proportions in the two arms. The intervention arm seems to have slightly higher proportion of women in younger age groups. The age factor has a lot of role to play in mobility status of women. 

 Also, it would add value to the description; if an account of 2 wave screening is given (the reason and circumstances for carrying out screening in 2 waves). 

 Is the sampling strategy followed the same as given in Mathews 2015?

Is the work clearly and accurately presented and does it cite the current literature?

Yes

If applicable, is the statistical analysis and its interpretation appropriate?

Yes

Are all the source data underlying the results available to ensure full reproducibility?

Yes

Is the study design appropriate and is the work technically sound?

Yes

Are the conclusions drawn adequately supported by the results?

Yes

Are sufficient details of methods and analysis provided to allow replication by others?

Partly

Reviewer Expertise:

Non Communicable Diseases (NCD), Nutrition

I confirm that I have read this submission and believe that I have an appropriate level of expertise to confirm that it is of an acceptable scientific standard.

# Peer review: Reviewer response for version 1

**DOI:** 10.21956/wellcomeopenres.18321.r43733

**Published:** 2021-05-17

**Authors:** Ravi Prasad Varma

**Affiliations:** 1 Achutha Menon Centre for Health Science Studies, Sree Chitra Tirunal Institute for Medical Sciences and Technology, Thiruvananthapuram, Kerala, India

The paper by Mathews E *et al.* describes the attempt by the investigators to increase the proportion of women in the intervention area in Thiruvananthapuram city who were physically active. Intervention and control areas were identified a priori. Women included in the study were identified to be sedentary through a screening survey which had two phases. A validated version of the Global Physical Activity Questionnaire (GPAQ) was used to measure physical activity – the outcome of interest. Metabolic equivalent task (MET) minutes per week levels were used to categorize participants into sedentary, moderate, or high levels of physical activity. The intervention is somewhat complex, with an amalgamation of community-level activities, group sessions, and peer-leader-led activities. Culturally appropriate forms of physical activity were identified and included in the study materials and interventions. The investigators conclude the intervention was effective in improving physical activity levels in women.

 Pertinent literature relevant to the research question has been reviewed rather well. The study design and participant selection are clearly described and appropriate. The intervention and control groups are comparable and the statistical analysis is suitable for the hypotheses tested and the conclusion drawn. Given the non-individual nature of the intervention, the groups formed per se may affect the intervention. That looks unlikely in this study as, in addition to similar physical activity levels at baseline (all were sedentary) there was a good chance of forming groups that were of similar demographics and living contexts. Given the gendered nature of the community, women’s access and roles in outdoor spaces could come under scrutiny from their families and the community. In this regard, the investigators’ efforts for community engagement and efforts for community ownership are very commendable and constitute good public health research practice.

 Envisioning process indicators based on the individual components of the interventions is tough in such a study and the investigators seem to have remained pragmatic in their approach. However, it is not clear how the investigators have conceptualized the dose-response relationship in this study. Interventions were tapered and physical activity decreased – whether that amounts to a dose-response relationship is questionable. I would recommend some caution while making statements suggesting implementation strength. Moreover, while it looks beyond the scope of this manuscript, I suggest exploring more clear-cut approaches to intervention strength if possible (Hargreaves *et al.* 2016 ^1^).

 The discussion portion has some other limitations too - the discussion on the possibility of measurement bias is mentioned but limited. There is also no mention of - (1) the physiological variables; (2) safety of the intervention/ possibility of harm - in the discussion. The lack of change in body mass index and waist circumference in physical activity interventions has been reported earlier and can be mentioned (Lamb *et al.* 2002 ^2^).

 I also suggest a few other minor edits/ corrections. The figure 1 legend may indicate that 'I' stands for intervention arm and 'C' for the control arm. Also, it may be indicated that the injury in the intervention group was independent of the intervention, if it were so. There are also a few minor typos – e.g. under the section “intense phase of intervention”, I think the authors meant NCDs in place of NCDSs.

 The main contribution of the paper is that there is very little published scientific literature on health promotion among women in low-and middle-income countries where women’s health is rarely envisaged beyond maternal health. Therefore I strongly recommend that this paper be accepted with minor revisions.

Is the work clearly and accurately presented and does it cite the current literature?

Yes

If applicable, is the statistical analysis and its interpretation appropriate?

Yes

Are all the source data underlying the results available to ensure full reproducibility?

Yes

Is the study design appropriate and is the work technically sound?

Yes

Are the conclusions drawn adequately supported by the results?

Yes

Are sufficient details of methods and analysis provided to allow replication by others?

Yes

Reviewer Expertise:

Epidemiology, Health care systems

I confirm that I have read this submission and believe that I have an appropriate level of expertise to confirm that it is of an acceptable scientific standard.

## References

[1] : Measuring implementation strength: lessons from the evaluation of public health strategies in low- and middle-income settings. *Health Policy Plan*.2016;31(7) : 10.1093/heapol/czw001 860-7 10.1093/heapol/czw001 26965038PMC4977426

[2] : Can lay-led walking programmes increase physical activity in middle aged adults? A randomised controlled trial. *J Epidemiol Community Health*.2002;56(4) : 10.1136/jech.56.4.246 246-52 10.1136/jech.56.4.246 11896130PMC1732126
